# Stable intronic sequence RNAs (sisRNAs) are selected regions in introns with distinct properties

**DOI:** 10.1186/s12864-020-6687-9

**Published:** 2020-04-07

**Authors:** Jing Jin, Ximiao He, Elena Silva

**Affiliations:** 1 Department of Physiology, School of Basic Medicine, Tongji Medical College, Huazhong University of Science and Technology, Wuhan, 430030 Hubei China; 2 Department of Biology, Georgetown University, 37th and O Sts, NW, Washington DC, 20057 USA

**Keywords:** sisRNA, Intron, Non-coding RNA, Oocyte, Transcription, *Xenopus*

## Abstract

**Background:**

Stable introns and intronic fragments make up the largest population of RNA in the oocyte nucleus of the frog *Xenopus tropicalis*. These stable intronic sequence RNAs (sisRNAs) persist through the onset of zygotic transcription when synchronous cell division has ended, and the developing embryo consists of approximately 8000 cells. Despite their abundance, the sequence properties and biological function of sisRNAs are just beginning to be understood.

**Results:**

To characterize this population of non-coding RNA, we identified all of the sisRNAs in the *X. tropicalis* oocyte nucleus using published high-throughput RNA sequencing data. Our analysis revealed that sisRNAs, have an average length of ~ 360 nt, are widely expressed from genes with multiple introns, and are derived from specific regions of introns that are GC and TG rich, while CpG poor. They are enriched in introns at both ends of transcripts but preferentially at the 3′ end. The consensus binding sites of specific transcription factors such as Stat3 are enriched in sisRNAs, suggesting an association between sisRNAs and transcription factors involved in early development. Evolutionary conservation analysis of sisRNA sequences in seven vertebrate genomes indicates that sisRNAs are as conserved as other parts of introns, but much less conserved than exons.

**Conclusion:**

In total, our results indicate sisRNAs are selected intron regions with distinct properties and may play a role in gene expression regulation.

## Background

RNA is one of the three major macromolecules essential for living organisms and consists of three major types: messenger RNA (mRNA), transfer RNA (tRNA), and ribosomal RNA (rRNA). Whereas mRNAs code for proteins [1], tRNAs and rRNAs are non-coding RNAs that play essential roles in protein translation [2, 3]. In the past 15 years, several additional types of non-coding RNAs have been shown to play important roles in regulation of gene expression [4], including microRNAs (miRNA, 21–22 nt) [5], small interfering RNAs (siRNA, 20–25 nt) [6], and Piwi-interacting RNAs (piRNA, 29–30 nt) [7]. While these regulatory non-coding RNAs are usually very small (< 50 nt), there are also the long non-coding RNAs (lncRNA), which are longer than 200 nucleotides [8], and most recently identified, the stable intronic sequence RNAs (sisRNAs), the majority of which are several hundreds of nucleotides [9, 10].

In higher eukaryotes, the majority of protein-coding genes have one or more non-coding introns interspersed within the coding sequence that are spliced from the primary transcript [11]. The spliced introns are primarily in the form of a lariat in which the 5′ end is linked to the 3’ acceptor splice site. These lariats are debranched into a linear form and then degraded rapidly in most cases [12, 13]. The majority of intron fragments are believed to be unstable, with a few exceptions [14–17]. Recently, a number of sisRNAs were identified in the oocyte nucleus (germinal vesicle or GV) of *Xenopus tropicalis* [9] and later in the oocyte cytoplasm [10]. Although less stable than cytoplasmic mRNA, the sisRNAs are very stable. Transcription inhibition studies revealed that they are stable for at least 2 days and RNA sequencing analysis demonstrated that they are transferred to the egg upon GV breakdown and persist until at least the blastula stage [9]. These sisRNAs are usually only a portion of the full intron and while nuclear sisRNAs are present as either linear or lariat molecules, most cytoplasmic sisRNAs are in lariat form [9, 10]. Thus far, sisRNAs have also been found in human, mouse, chicken, zebrafish, the Epstein-Barr virus and *Drosophila melanogaster* [9, 18–22]. Little is known about the sequence properties and biological function of these abundant sisRNAs although recently, sisR-1 and sisR-4, have been shown to participate in a feedback loop to modulate their parental gene expression in *Drosophila* [20, 22–24].

To further characterize sisRNAs, we identified all sisRNAs in the *X. tropicalis* genome using the published high-throughput sequencing data of RNA from the GV [9]. We then determined the average length of sisRNAs, their distribution in genes, sequence composition, transcription factor binding site (TFBS) enrichment, gene ontology and evolutionary conservation. Here we show that sisRNAs are most widely expressed from genes with multiple introns, enriched in GC and TpG. They are enriched in introns at both ends of transcripts but preferentially at the 3′ end. They also contain specific transcription factor binding sites (TFBS), which supports recent findings that sisRNAs play a role in the regulation of gene expression.

## Results

### Genome-wide identification of sisRNAs in *Xenopus tropicalis*

**Fig. 1:**
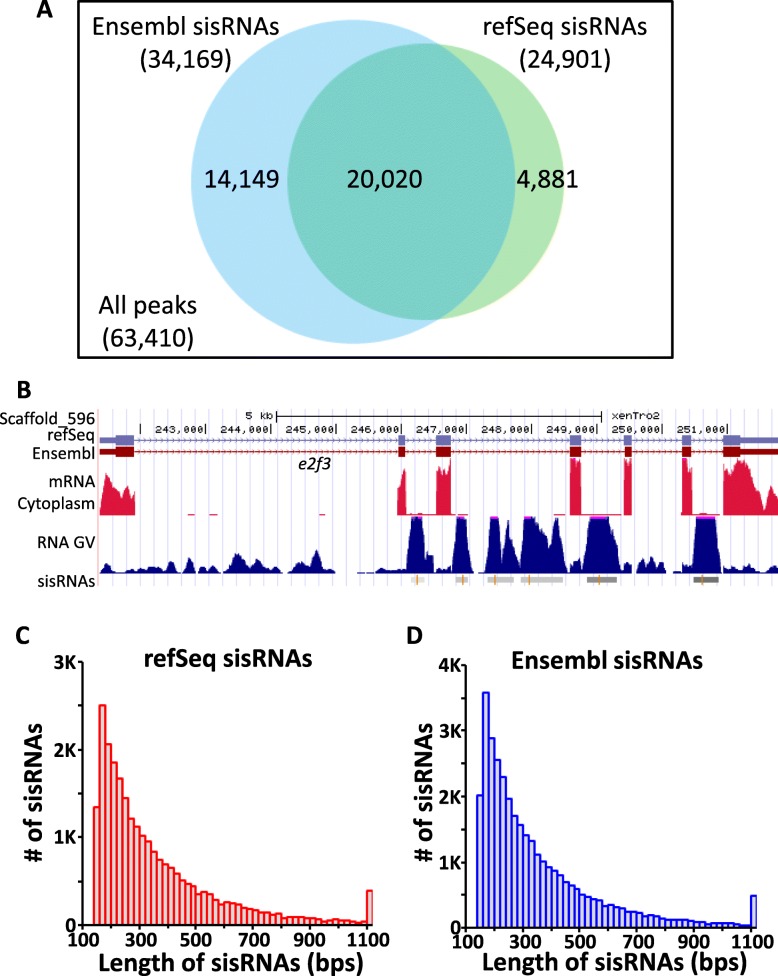
sisRNAs are identified from GV RNA-seq data according to Ensemble and RefSeq gene sets in *X. tropicalis.* **a** Venn diagram of peaks called by MACS with FDR=0.01 and determined to be sisRNAs according to refSeq and Ensembl gene sets. **b** UCSC screenshot of identified sisRNAs in the gene E2F3. Red and blue blocks indicate exons, red peaks indicate mRNA detected in the cytoplasm, blue peaks indicate RNA detected in the GV, grey blocks indicate sisRNAs identified, orange lines indicate the summits of sisRNA peaks. **c-d** Histograms of length distributions for (**c**) refSeq and (**d**) Ensembl sisRNAs

To study the sequence characteristics of sisRNAs, the high-throughput sequencing data of RNA (RNA-seq) from the GV of the frog *Xenopus tropicalis* [9] was used to detect sisRNA peaks with the model based analysis for ChIP-seq (MACS) algorithm [25]. We identified a total of 63,410 RNA peaks by a more stringent criterion (FDR = 0.01) (Fig. 1a, Table 1) and compared the location of each RNA peak to exons and introns to identify the true sisRNA peaks (i.e. those from spliced introns that did not cross intron–exon boundaries.) Two widely used and primarily manually annotated gene sets, RefSeq (9448 protein-coding genes) and Ensembl (28,967 protein-coding genes), were used as references to identify the sisRNA peaks. Twenty-four thousand nine hundred and one sisRNAs were identified by refSeq genes (termed as refSeq sisRNAs), with a total length of ~ 9 Mbps, which accounts for 0.6% of the *X. tropicalis* genome (Fig. 1a, Table 1). Similarly, 34,169 sisRNAs were identified by Ensembl genes (termed as Ensembl sisRNAs), with a total length of ~ 12 Mbps, which accounts for 0.8% of the genome (Fig. 1a, Table 1). Together, a total of 20,020 peaks were identified by both gene sets (Fig. 1a), which represents ~ 80% of refSeq sisRNAs and ~ 60% of Ensembl sisRNAs respectively. The sisRNA length ranges from 160 bps to 2908 bps with the majority 200–500 bps long and an average of 363.6 bps and 356.6 bps for refSeq and Ensembl sisRNAs, respectively (Fig. 1b-d, Table 1). Taken together, two high quality datasets of genome-wide sisRNAs were generated for further investigation of sisRNA properties.

**Table 1:** sisRNAs identified in GV RNA-seq reads by MACS according to Ensemble and RefSeq genes in *Xenopus tropicalis*

Genome size: 1,513,925,492 bps	#	Total length	% in genome	Length distribuion			
				average	min	median	max
**All peaks by MACS (FDR=0.01)**	63,410	21,899,763	1.45%	345.4	160	271	2908
**sisRNAs by Ensembl genes (28,937)**	34,169	12,184,476	0.80%	356.6	160	283	2908
**sisRNAs by RefSeq genes (9448)**	24,901	9,054,977	0.60%	363.6	160	288	2908

### sisRNAs are widely expressed and preferentially located in genes with multiple introns

**Fig. 2:**
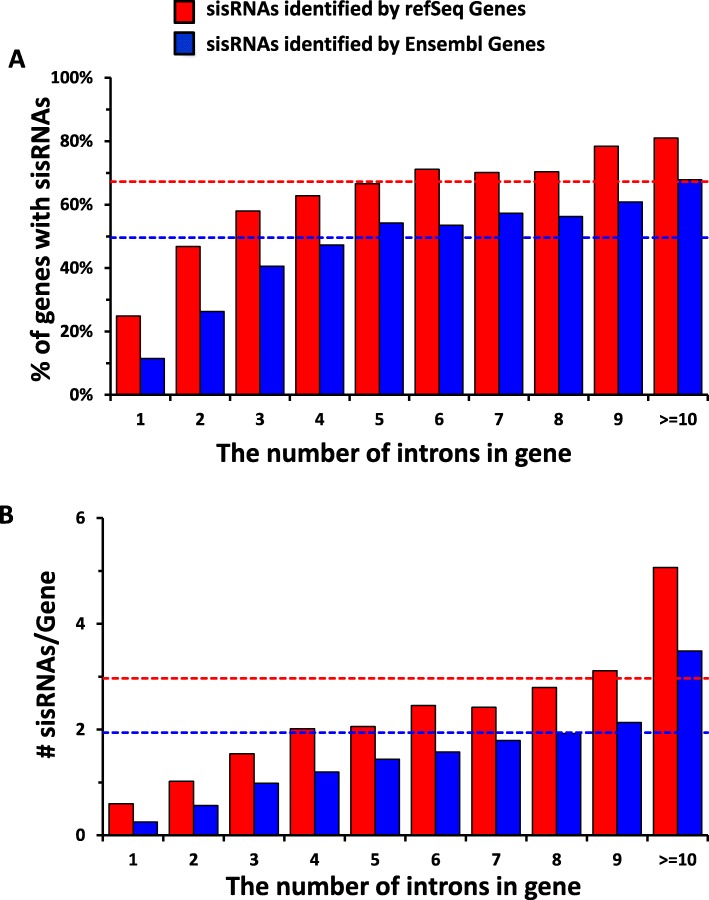
sisRNAs are highly expressed from genes with multiple introns. **a-b** Histograms show (**a**) the percentage of genes with sisRNAs, and (**b**) the average number of sisRNAs per gene in refSeq (red) and Ensembl (blue) genes. Genes are grouped by the intron number. The dashed line indicates the average value for all genes

To determine the number of genes with sisRNAs, we first divided the genes of each dataset into 10 groups according to the number of introns in a gene. Genes without introns were excluded from further analysis. From the 9448 RefSeq genes, 93.8% (8864) have multiple exons while only 6.2% (583) of the genes have a single exon (Tables 2). Our data show that the more introns a gene has, the more likely the gene generates sisRNAs. While 24.9% of genes with a single intron have sisRNAs located in their intron, the possibility that a gene with 5 introns has a sisRNA increased to 66.6%. When a gene has 10 or more introns the possibility increased to 81.0% (Fig. 2a). On average, 67.3% of Refseq genes (5965/8864) with introns produce sisRNAs (Fig. 2a, Table 2).

**Table 2:** sisRNAs in RefSeq genes

#Introns/gene	#	# of total introns	# of genes with sisRNAs	% of genes with sisRNAs	# of sisRNAs	#sisRNAs/Gene	#sisRNAs/Intron
0	584	–	–	–	–	–	–
1	583	583	145	24.9%	347	0.60	0.60
2	729	1458	341	46.8%	743	1.02	0.51
3	812	2436	471	58.0%	1254	1.54	0.51
4	777	3108	488	62.8%	1564	2.01	0.50
5	716	3580	477	66.6%	1472	2.06	0.41
6	683	4098	486	71.2%	1677	2.46	0.41
7	599	4193	420	70.1%	1450	2.42	0.35
8	580	4640	408	70.3%	1621	2.79	0.35
9	506	4554	397	78.5%	1575	3.11	0.35
>=10	2879	45,110	2332	81.0%	14,581	5.06	0.32
Total	9448	73,760	5965	67.3%	26,284	2.97	0.36

We also calculated the average number of different sisRNAs per gene. We observed that the average sisRNA number increases with intron number (the average number of introns per gene is 7.8 in *Xenopus tropicalis*). For example, genes with a single intron have an average of 0.60 sisRNAs, genes with 5 introns have an average of 2.06 sisRNAs, and genes with 10 or more introns have an average of 5.06 sisRNAs (Fig. 2b, Table 2). On average, there are 2.97 sisRNAs per gene and 0.36 sisRNAs per intron (Table 2). We also compared the intron length to the number of sisRNAs for each intron, and it indicates that longer introns tend to have more sisRNAs (R=0.72, Supplementary Fig. 1A-B). Next, we compared the total length of introns (Supplementary Fig. 1C) or normalized intron number per Mb (Supplementary Fig. 1D) to the number of sisRNAs for each gene. The results indicate that the number of sisRNAs is well correlated with the total length of introns (R=0.87) rather than the number of introns (R=0.09) in a gene. We observed a similar result for Ensembl genes (Fig. 2, Table 3).

**Table 3:** sisRNAs in Ensembl genes

#Introns/gene	#	# of total introns	# of genes with sisRNAs	% of genes with sisRNAs	# of sisRNAs	#sisRNAs/Gene	#sisRNAs/Intron
0	2654	–	–	–	–	–	–
1	3600	3600	415	11.5%	911	0.25	0.25
2	2461	4922	648	26.3%	1383	0.56	0.28
3	1986	5958	807	40.6%	1951	0.98	0.33
4	1791	7164	847	47.3%	2140	1.19	0.30
5	1696	8480	919	54.2%	2439	1.44	0.29
6	1543	9258	826	53.5%	2431	1.58	0.26
7	1458	10,206	835	57.3%	2613	1.79	0.26
8	1372	10,976	772	56.3%	2637	1.92	0.24
9	1260	11,340	767	60.9%	2687	2.13	0.24
>=10	9146	162,302	6208	67.9%	31,887	3.49	0.20
Total	28,967	234,206	13,044	49.6%	51,079	1.94	0.22

### The sisRNA sequences are GC and TpG rich

**Fig. 3:**
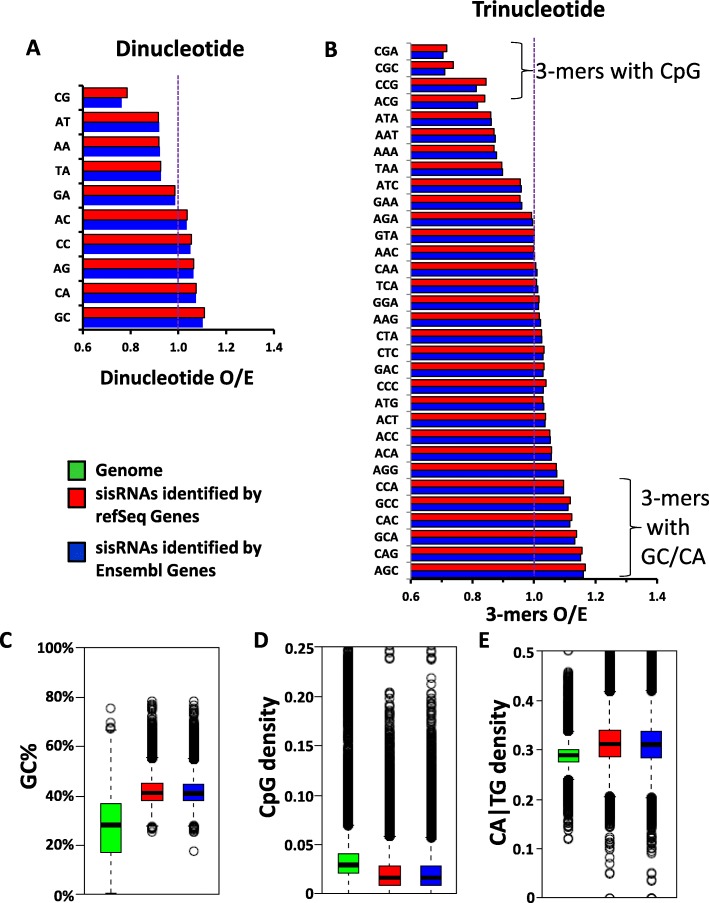
The sequences of sisRNAs have higher GC and TG content, while CpG poor compared to the genome. **a**-**b** The ratio of observed/expected (O/E) for the occurrence of dinucleotide (**a**) and trinucleotide (**b**) combinations in sisRNAs identified by refSeq genes (red) and by Ensembl genes (blue). **c**-**e** Boxplots show the GC% (**c**), CpG density (**d**) and CA/TG density (**e**) of the genome (green) compared to sisRNAs identified by refSeq (red) and Ensembl (blue) genes

To investigate the base-pair composition in sisRNAs, we first calculated the prevalence of all 10 unique dinucleotides in sisRNAs and in the *X. tropicalis* genome. We observed that sisRNAs as compared to the *X. tropicalis* genome are rich in AC, CC, AG, CA and GC, while poor in CG, AT, AA, TA and GA, for both RefSeq- and Ensembl- identified sisRNAs (Fig. 3a). We then extended the calculation to trinucleotides. As we expected, sisRNAs are rich in CCA, GCC, CAC, GCA, CAG, AGC, which are all trinucleotides containing GC or CA|TG, the dinucleotides shown to be enriched in sisRNAs (Fig. 3b). sisRNAs are very poor in CGN, which are trinucleotides with CpG, and also poor in ATA, AAT, AAA and TAA (Fig. 3b). Generally speaking, CpG rich regions (e.g. CpG islands) are G+C rich and CpG poor regions are A+T rich. Interestingly, we observed that sisRNAs are CpG poor while GC rich.

We then calculated the GC content, CpG density and CA|TG density for each sisRNA as well as each scaffold in the whole genome (Fig. 3c-e). Compared to the *X. tropicalis* genome, sisRNAs have a higher GC content (Fig. 3c), a lower CpG density (Fig. 3d) and a higher CA|TG density (Fig. 3e). As a result, in terms of base-pair compositions, sisRNAs are different from the genome, with a different prevalence of dinucleotides, trinucleotides, and a lower CpG density, higher CA|TG density and GC content. In other words, sisRNAs are unique regions of the genome with their own properties. The results for RefSeq sisRNAs and Ensembl sisRNAs are nearly identical (Fig. 3), providing strong support for our results.

### The sisRNAs are specific regions of the introns

**Fig. 4:**
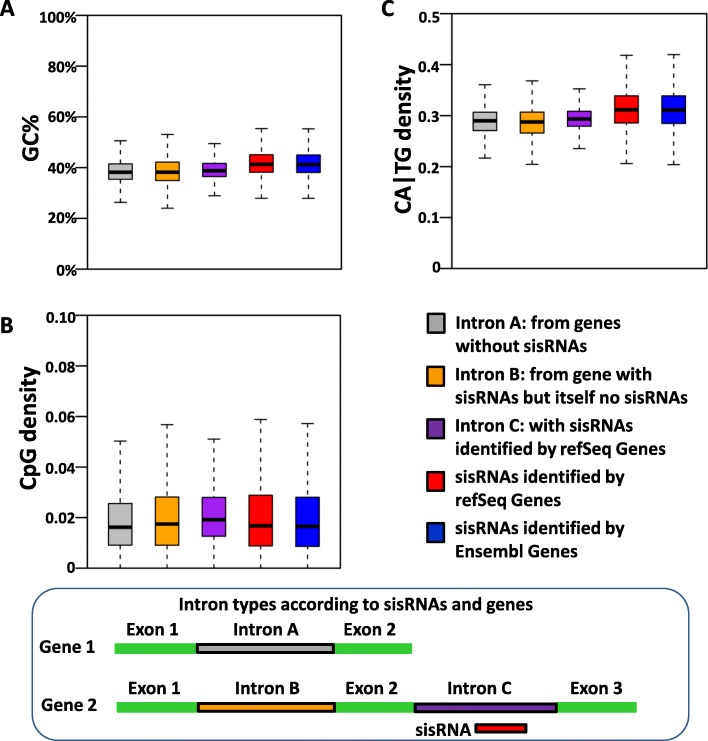
sisRNAs are specific regions of the introns. Boxplots of the comparisons of GC% (**a**), CpG density (**b**) and CA|TG density (**c**) of the introns from genes without any sisRNA (grey), introns without sisRNA from host genes with sisRNAs (orange), and introns with sisRNAs (purple) to sisRNAs identified by refSeq (red) and Ensembl (blue) genes

Since sisRNAs are derived from regions of introns, and introns have a distinct base-pair composition as compared to the whole genome, we expect sisRNAs to also have a sequence composition different from the whole genome. However, it remains unclear whether sisRNAs are randomly distributed in introns or in specific regions of the intron. GC content (G+C%), CpG density and CA|TG density are widely used and important parameters to analyze sequences characteristics. Thus, we calculated these parameters for each intron with/without sisRNAs and compared these results with sisRNAs alone. The total GC content of the introns with sisRNAs (40.59%) is higher (*p*< 0.001, t-test) than the genome (40.07%). The GC content in sisRNAs alone (41.92% and 41.79% for RefSeq and Ensembl sisRNAs, respectively) is higher than that of the introns (p< 0.001, t-test) (Fig. 4a, Table 4). The CpG density of introns when compared to the whole genome is poor (Fig. 4b). Interestingly, introns with sisRNAs have a higher CpG density than both introns without sisRNAs and sisRNAs alone (Fig. 4b). While the CA|TG density in introns is very similar to the genome, sisRNAs have a higher CA|TG density than introns with sisRNAs (Fig. 4c). Thus, the sisRNAs are in regions of the introns with higher CA|TG density and GC%. We further divided the introns without sisRNAs into two groups according to whether the host gene is with/without sisRNAs: Intron A (the host gene without sisRNAs) or Intron B (the host gene has sisRNAs but the intron itself is without sisRNAs). We found that intron A and intron B are very similar, thus sisRNAs are closely associated with the introns from which they originate. Taken together, these results reveal that sisRNAs are specific regions of introns with distinct sequence compositions.

**Table 4:** Base compositions in whole genome, sisRNAs and introns in *X. tropicalis*

Bases	Genome	RefSeq sisRNAs	Ensembl sisRNAs	Introns	Introns
				- sisRNAs	+ sisRNAs
**A**	29.97%	29.09%	29.14%	30.32%	29.74%
**C**	20.04%	20.92%	20.88%	19.66%	20.27%
**G**	20.04%	21.00%	20.91%	19.67%	20.32%
**T**	29.96%	28.99%	29.07%	30.36%	29.67%
**total**	1,359,400,017	9,027,876	12,146,831	117,363,589	105,176,094
**(G+C)%**	40.07%	41.92%	41.79%	39.32%	40.59%

### sisRNAs are enriched at both 5′ and 3′ end of transcripts, with a preference for the 3′ end

**Fig. 5:**
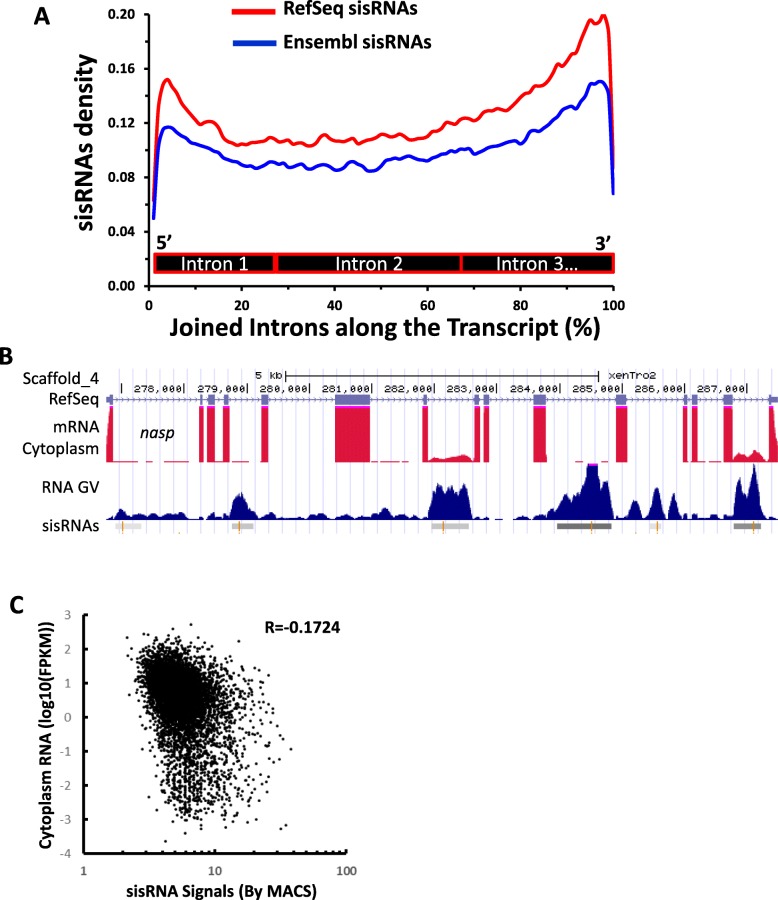
sisRNAs are enriched at both the start and the end of transcript, preferentially the 3′ end of transcript. **a** The density of sisRNAs in introns. For each transcript, all the introns are concatenated from 5′ to 3′ end, and then divided into 100 bins. **b** UCSC genome browser screenshot of sisRNAs distribution along the introns of the gene *nasp*. A higher number of sisRNAs are located at the 3′ end. **c** Scatter plot of sisRNA peak signals versus host gene expression level (FPKM)

After we observed that sisRNAs are in specific regions of introns, with unique base-pair compositions, we analyzed whether they are derived from specific introns along the gene. To study where sisRNAs are enriched, we concatenated all the introns for each gene along the transcript. We divided each joined intron transcript into 100 bins and identified the bins in which the sisRNAs are located. As shown in Fig. 5a, the sisRNAs are mostly enriched at the beginning (5′ end) and the end (3’ end) of the transcript with a preference for the 3′ end. An example is shown for the gene *nasp*: more sisRNAs were observed at the 3′ end (Fig. 5b). These results further confirmed that sisRNAs are not random sequences from the introns: they have distinct sequence compositions and are preferentially driven from the 3′ end of a transcript.

We next asked whether sisRNAs are derived from the 5′ and 3′ end of introns because these end introns have unique properties. To investigate this possibility, we divided all the introns from genes with more than 3 introns into 3 categories: 1^st^ − 5′ end introns (S), middle introns (M) and last − 3′ end introns (E). The results indicate that last − 3′ end introns are very similar to the middle intron, while the first − 5′ end introns have different compositions and are slightly longer (Supplementary Fig. 2). The properties of the first introns may be different because they sometimes overlap with the promoter regions, which have higher CpG, CA|TG density, and GC content. These results indicate that sisRNAs are not preferentially derived from 3′ end introns because these introns have unique properties, but instead some other mechanism of sisRNA production is involved.

Another possibility is that sisRNAs produced from 3′ end introns are remnants of gene transcription and splicing. To test this idea, we performed the analysis of correlation between the expression levels of host genes and sisRNAs. As shown in Fig. 5c, the expression levels of host genes (FPKM) and sisRNAs peak signals are negatively relevant (R=-0.1724), which does not support sisRNAs as remnants of gene transcription and splicing that have not been cleaned from cells. Thus, the stability of sisRNAs is regulated independently from the host mRNAs. Taken together, sisRNAs are most enriched in the 3′ end of introns, and the mechanism for this enrichment remains to be investigated.

### Specific TFBSs are enriched in sisRNA-producing introns

**Fig. 6:**
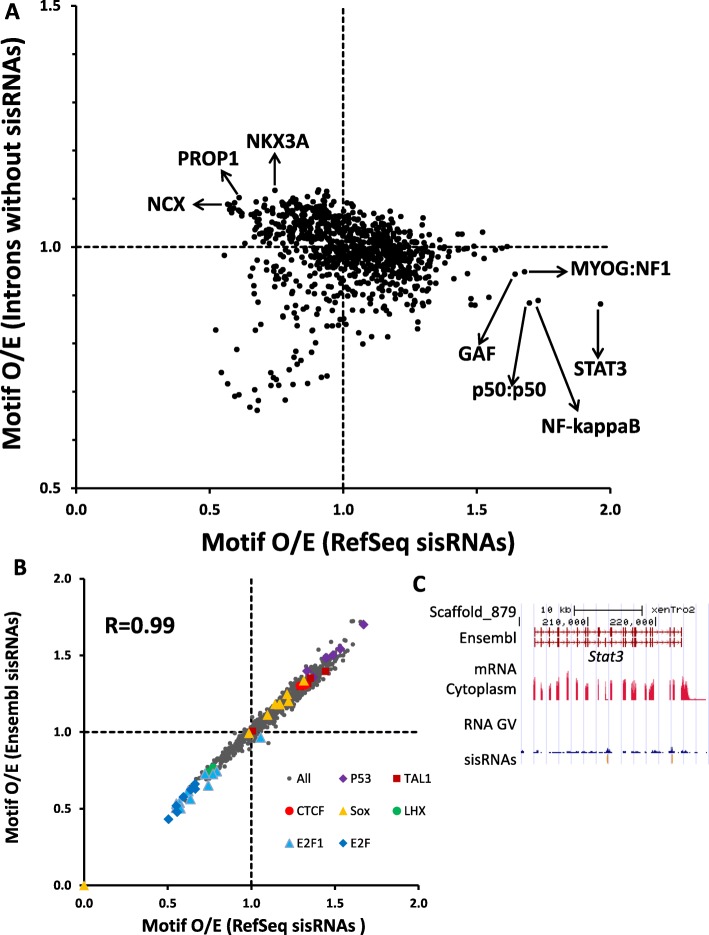
Specific TFBSs are enriched in sisRNAs. The enrichment of TFBS motifs was plotted for (**a**) the introns without any sisRNAs versus the sisRNAs identified by RefSeq genes, and (**b**) the RefSeq sisRNAs versus the Ensembl sisRNAs. **c** UCSC genome browser screenshot of Stat3 shows it is highly expressed in the cytoplasm

Because of the specific sequence properties and preference for introns on the ends of a gene, we performed an enrichment calculation of transcription factor binding sites (TFBS) in sisRNAs to study the potential functions of sisRNAs. We searched for enriched DNA motifs among the 935 position weight matrices (PWMs) collected from the TRANSFAC databases [26] in sisRNAs and in introns without sisRNAs (Fig. 6). We also calculated the motif enrichment in both RefSeq and Ensembl sisRNAs and the enrichments in two datasets are nearly identical (R=0.99), indicating the calculation is robust and the quality of identified sisRNAs is high (Fig. 6b). Stat3, NF-κB, p50:p50, MYOG:NF1 and GAF consensus sites are enriched in sisRNAs but depleted in introns without sisRNAs (Fig. 6a). Stat3 (signal transducer and activator of transcription 3) is a member of the STAT protein family, functions as a transcriptional activator [27], and is highly expressed in *X. tropicalis* (Fig. 6c). NF-κB (nuclear factor kappa-light-chain-enhancer of activated B cells) is a protein complex that controls transcription [28]. p50 is the mature NF-κB subunit, which has no intrinsic ability to activate transcription and has been proposed to act as a transcriptional repressor when binding with κB elements as homodimers (p50:p50) [29]. Myogenin (MYOG) is one of four muscle-specific basic helix-loop-helix regulatory factors involved in controlling myogenesis [30], and NF-1 (Neurofibromin 1) is a negative regulator of the Ras signal transduction pathway [31] and is required for skeletal muscle development [32]. The GAGA factor (GAF) is one of a few transcription factors that can regulate transcription at multiple levels: depending on its target genomic location, it can act as either activator or repressor [33]. We also observed that some TFBS, such as Ncx, Prop1 and Nkx3a, are depleted in sisRNAs while slightly enriched in introns without sisRNAs (Fig. 6a). These results suggest that specific TFBS involved in transcription regulation, either activation or suppression, are enriched in sisRNA-producing introns, which imply that sisRNAs may play a functional role in transcriptional regulation.

### The GO terms nucleotide binding, RNA binding and ATP binding are enriched in genes with sisRNAs

To further investigate the role of sisRNAs, we asked whether the genes with sisRNAs share any function. GO (Gene Ontology) enrichment analysis [34] of the 5419 RefSeq genes with sisRNAs indicated that 13.9% of the genes are involved in nucleotide binding, 3.2% of the genes are involved in RNA binding and 7.9% genes are involved in ATP binding (Table 5). The other enriched gene sets included RNA processing, mRNA metabolic process and translation (Table 5). These results suggested that sisRNAs might have a potential biological function involved in gene regulation and metabolism.

**Table 5:** The gene function analysis by DAVID from the 5419 genes with sisRNAs identified by RefSeq genes

Category	Term	Count	%	P-Value
GOTERM_MF_FAT	nucleotide binding	690	13.9	3.30E-24
GOTERM_MF_FAT	RNA binding	161	3.2	6.10E-17
GOTERM_MF_FAT	adenyl ribonucleotide binding	396	8	2.10E-16
GOTERM_MF_FAT	nucleoside binding	420	8.4	2.10E-16
GOTERM_MF_FAT	adenyl nucleotide binding	418	8.4	2.20E-16
GOTERM_MF_FAT	ATP binding	395	7.9	2.40E-16
GOTERM_MF_FAT	purine nucleoside binding	418	8.4	2.90E-16
GOTERM_MF_FAT	purine nucleotide binding	545	11	3.30E-14
GOTERM_MF_FAT	ribonucleotide binding	523	10.5	4.60E-14
GOTERM_MF_FAT	purine ribonucleotide binding	523	10.5	4.60E-14
GOTERM_BP_FAT	RNA processing	119	2.4	1.30E-12
GOTERM_CC_FAT	ribonucleoprotein complex	148	3	2.20E-12
GOTERM_CC_FAT	intracellular non-membrane-bounded organelle	312	6.3	8.50E-11
GOTERM_CC_FAT	non-membrane-bounded organelle	312	6.3	8.50E-11
GOTERM_BP_FAT	mRNA metabolic process	63	1.3	1.20E-10
GOTERM_BP_FAT	modification-dependent macromolecule catabolic process	101	2	6.60E-10
GOTERM_BP_FAT	modification-dependent protein catabolic process	101	2	6.60E-10
GOTERM_BP_FAT	cellular macromolecule catabolic process	117	2.4	1.80E-09
GOTERM_BP_FAT	mRNA processing	57	1.1	2.20E-09
GOTERM_BP_FAT	protein catabolic process	113	2.3	3.30E-09
GOTERM_BP_FAT	cell division	58	1.2	6.40E-09
GOTERM_BP_FAT	translation	138	2.8	8.80E-09

### The sisRNAs are as evolutionary conserved as introns, and much less than exons

**Fig. 7:**
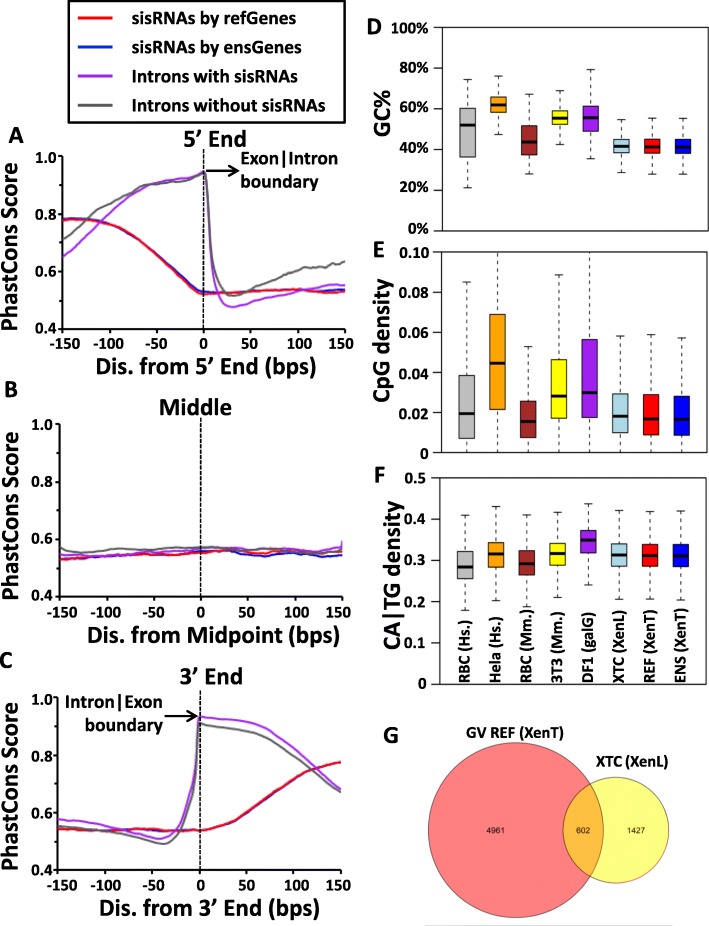
sisRNAs are as evolutionary conserved as introns, but much less than exons. Average PhastCons conservation scores of sisRNAs and introns for upstream and downstream (±150-bps) relative to (**a**) 5′ end, (**b**) midpoint, and (**c**) 3′ end. Boxplots of the comparisons of GC% (**d**), CpG density (**e**) and CA|TG density (**f**) of the human red blood cells (grey), human Hela cells (orange), mouse red blood cells (brown), mouse 3 T3 cells (yellow), chicken DF1 cells (purple), and *Xenopus laevis* XTC cells (light blue) cytoplasmic sisRNAs, to sisRNAs identified by refSeq (red) and Ensembl (blue) genes. **g** Venn diagram shows the overlap of host genes with sisRNAs identified by RefSeq genes in *Xenopus tropicalis* GV and host genes with cytoplasmic sisRNAs in *Xenopus laevis* XTC

Our data indicate that sisRNAs are in specific regions of introns, contain TFBSs, and are in the introns of genes involved in nucleotide, ATP and RNA binding. To investigate whether these sisRNAs are conserved across species, we determined the PhastCons conservation score for sisRNAs and introns (Fig. 7). The PhastCons scores [35] are calculated based on multiple alignments of 6 vertebrate genomes (zebrafish, chicken, opossum, rat, mouse and human) with *X. tropicalis*. As expected, the boundary of exon and intron has the highest conservation score (Fig. 7a, c). Although sisRNAs are as conserved as other intron regions, they are still much less conserved than exons, suggesting they might not be conserved among species, which was also shown in a recent study [10].

An alternative approach to evaluate the conservation of sisRNAs is to compare the sisRNAs in different cell types from various species. We obtained the cytoplasm sisRNAs data recently published [21], which contains several species and cell types, including human red blood cells, Hela cells, mouse red blood cells and 3 T3 cells, chicken DF1 cells, and *Xenopus laevis* XTC cells. We compared the GC content, CpG density, and CA|TG density among these cytoplasm sisRNAs to the refSeq and Ensembl sisRNAs that we identified (Fig. 7d-f). We found that between different cell types in the same species, for example, human red blood cells and Hela cells, the GC content, CpG density and CA|TG density of these sisRNAs are quite different from each other (Fig. 7d-f). While for the same cell types between different species, for example, human red blood cells and mouse red blood cells, some sequence composition similarities exist (Fig. 7d-f). These results indicate that sisRNAs are cell type specific. Although sequences of sisRNAs may not be so conserved among species, the sequence compositions are similar.

The *X. laevis* XTC sisRNAs are very similar to *X. tropicalis* GV sisRNAs in terms of sequence composition. We next asked how many common host genes with sisRNAs exist between the two species. We compared 5563 unique genes host sisRNAs in GV of *X. tropicalis* to 2029 unique genes which have sisRNAs in *X. laevis* XTC cells, there are only 602 genes overlapped and this chance is even lower than random selection (Fig. 7g). Among these 602 genes, the number of sisRNAs from the same intron is less than 50 (data not shown). Overall, our calculation suggested there may not be positional or sequence conservation between sisRNAs, which is also supported by other recent work [10].

We also performed GO (Gene Ontology) enrichment analysis for the genes host sisRNAs in each cell type (Supplementary Fig. 3A-F). In chicken DF1 cells, the sisRNAs are not enriched in any gene sets (Supplementary Fig. 3E). Even though sisRNAs are enriched in some gene sets in the other 5 cell types, there are no common enriched gene sets present in all 5. The most common enriched gene set is related to sister chromatid segregation or nuclear division, which is observed in human RBC, Hela, mouse RBC, and *X. laevis* XTC cells (Supplementary Fig. 3A-C, F). Another common enriched biological process is covalent chromatin modification, which is observed in human RBC, mouse RBC and 3 T3 cells (Supplementary Fig. 3A, C-D). It will be interesting to test whether sisRNAs play functional roles in cell division or chromatin organization.

## Discussion

### The properties of sisRNAs in *Xenopus tropicalis*

The biological function of sisRNAs in the *Xenopus* oocyte nucleus is unclear but of particular interest especially in light of their stability and abundance. The majority of zygotic transcription in the *Xenopus* embryo begins at the midblastula transition (MBT), after the 12^th^ cell division. The transcription rate must be extremely high in the oocyte and the transcripts must be very stable to allow a sufficient amount of RNA to be deposited in the cytoplasm such that the RNA:DNA ratio is not significantly depleted as the cells divide before MBT [36, 37]. In this case, the sisRNAs could simply be the byproducts of universal stable RNA transcripts [9]. However, we found that sisRNAs are not randomly distributed in introns, but rather in specific regions of the intron, with a unique sequence composition, indicating that these sisRNAs are selected to be stable and abundant and thus are very likely to have a relevant biological function in the *Xenopus* oocyte and early embryonic development.

Recently, 9000 sisRNAs have been found in the cytoplasm with about half of these confirmed as lariat molecules [10]. These sisRNAs are only derived from a relatively small number of specific introns [10], which further confirmed our observations that sisRNAs are not random sequences but specific regions of introns. Besides *Xenopus*, numerous circular intronic sequences have been identified in cultured human cell lines (Hela and H9) [19] implying that they are widely expressed in many different species and may have a significant biological role [22].

### The association of sisRNA-producing introns with Stat3

We showed that consensus TFBS of Stat3 is the most enriched motif in the introns from which sisRNAs are generated, and observed that Stat3 is highly expressed in *Xenopus tropicalis* oocytes [9]. A recent microarray study showed that Stat3 is expressed at stage 2 and peaks at stage 8 and is still detectable as late as stage 33 in both *X. tropicalis* and *X. laevis* [38]. It has been widely reported that Stat3 can bind to intronic regions to regulate gene expression. For example, the expression of BCL3 is induced by IL-6 via Stat3 binding to intronic enhancer HS4 [39]. The signaling factor, WNT5A is an evolutionarily conserved target of the Stat3 signaling cascade based on 11-bp-spaced tandem Stat3-binding sites within intron 4 of human, chimpanzee, cow, mouse and rat WNT5A orthologs [40]. Stat3 binding to the introns of Foxp3, RORα, RORγt, and IL-6Rα have also been reported [41–43]. Analysis of ~ 75,000 Stat3 binding sites identified by chromatin immunoprecipitation (ChIP)-seq in a transformed human breast cell line revealed that most Stat3 binding sites are located within introns [44]. In total, these data indicate a role for Stat3 in the regulation of expression of the genes from which sisRNAs arise. It is also possible that specific TF interactions are involved in the biogenesis of sisRNAs. Both of these seem more likely considering STAT3 is a TF that binds DNA and RNA [45]. A recent study showed that lncRNA directly binds STAT3 in the cytoplasm of human dendritic cells (DC), thereby preventing dephosphorylation of STAT3 by SHP1, and controlling the differentiation of DC [46]. Another study demonstrated an interaction between STAT3 and circular RNAs [47], which may be a type of sisRNA. Taken together, future experiments can be designed to test if sisRNAs interact with STAT3 and if their formation is dependent on STAT3.

### The evolutionary origin of sisRNAs

DNA methylation at the 5′ position of cytosine (5mC), primarily in CpG context, is observed in nearly every vertebrate examined, including *Xenopus tropicalis* [48]. 5mC can deaminate to thymine 10–50 times faster than the mutation rate of other nucleotides [49]. Deamination of 5mC caused the high depletion of the CpG dinucleotide in mammalian genomes [50], and as a result, TG (the deamination product of 5mCG) is the most abundant dinucleotide in vertebrates [51]. The unmethylated CGs [52] tend to be clustered together into CG islands (CGI) [53]. As a result, CpG rich regions (CG islands) have a higher GC content, whereas CpG poor regions have a lower GC content. We observed that CpG is depleted in sisRNAs as compared to the *X. tropicalis* genome, and as expected, TG is enriched. Interestingly, we observed higher GC content in sisRNAs, which suggested that the base composition of sisRNAs is not merely the consequence of deamination of 5mC, there must be other mechanisms playing a role in the evolutionary origin of sisRNAs. A recent study showed that exons of lncRNA loci also have a high GC content due to purifying selection [54], thus it is possible that sisRNAs share some evolutionary properties with lncRNAs. Surprisingly, the CpG density in sisRNAs alone is very similar to introns without sisRNAs, and lower than that of introns with sisRNAs. This suggests that sisRNAs are surrounded by regions with a high CpG level. In other words, although sisRNAs themselves are CpG poor, they are derived from introns with high CpG density, and higher CpG density in the introns may be indicative of producing sisRNAs.

We also assessed the evolutionary conservation of the *X. tropicalis* sisRNA sequences in six other vertebrate genomes, including zebrafish, chicken, opossum, rat, mouse and human. Our results indicated that sisRNAs are not conserved: sisRNAs are as conserved as other parts of introns, but much less conserved than the exons. Cytoplasmic sisRNAs have been identified recently in *X. tropicalis* oocytes and these sisRNAs are only derived from a relatively small number of specific introns [10], it is worth noting that these sisRNAs are also not conserved.

Thus far sisRNAs are identified in human, mouse, chicken, zebrafish, *Xenopus* and *Drosophila*, implying that sisRNAs might be widely expressed in many other species [21, 22]. Considering that sisRNAs are not conserved, if sisRNAs do have some biological functions such as gene regulation, it might be cell type and species-specific.

In conclusion, our results suggest that sisRNAs are not transcribed from random part of introns but specific regions with distinct properties. The sisRNAs are GC rich while CpG poor, and preferentially enriched in the 3′ end of the mRNA transcript. Specific TFBSs involved in gene regulation are enriched in the regions from which sisRNAs arise, suggesting an association with specific proteins, such as Stat3, and further experiments are required to investigate this association. With more and more sisRNA data available in different species, the potential biological functions of sisRNAs would hopefully to be revealed soon.

## Methods

### Dataset generation

The GV and cytoplasmic RNA-seq data were obtained from the Gall lab [9]. sisRNA sequences from human red blood cells, Hela cells, mouse red blood cells, mouse 3 T3 cells, chicken DF1 cells, *X. laevis* XTC cells were obtained from [21]. All the sequencing data were produced by the Gall lab, and acquisition and culturing of cells are described in detail [21]. Briefly, human HeLa cells, mouse 3 T3 cells, chicken DF1 cells and XTC cells are cell lines cultured in different mediums [21]; human RBCs were purchased from the Interstate Blood Bank (through Zen-Bio, Inc.); mouse tissues were provided by the Bortvin laboratory, Department of Embryology, Carnegie Institution for Science, Baltimore, MD [21]. The dataset of refSeq and Ensembl genes, PhastCons conservation scores, as well as the genome sequences of *Xenopus tropicalis* were downloaded from the University of California Santa Cruz Genome Bioinformatics website (http://genome.ucsc.edu/) [55]. The reference *X. tropicalis* genome assembly was xenTro2 (assembly version 4.1). Exons, introns, 5’UTRs, and 3’UTRs for refSeq genes and Ensembl genes were determined using UCSC annotations. UCSC genome browser screen shots were generated using custom tracks of the UCSC web site (https://genome.ucsc.edu/).

### Identification of sisRNAs in germinal vesicles

The Model-Based Analysis of ChIP-seq algorithm (MACS) [25] was used for detecting sisRNAs peaks by analyzing germinal vesicle (GV) RNA-seq data [9]. First, we identified all 63,410 peaks of the GV RNA-seq data with default parameters, but a more stringent FDR=0.01 (default is 0.05). We then determined the location of each peak relative to exon and intron annotation for refSeq and Ensembl genes, respectively. A peak was identified as a sisRNA if it was located within an intron and did not overlap with an exon. We set the cut-off length of 150-bps for the sisRNAs to rule out the potential contamination of short non-coding RNAs (e.g. miRNA, siRNA, piRNA). In this way we identified 24,901 and 34,169 sisRNAs for refSeq and Ensembl annotated genes, respectively.

### Calculation of sisRNA density along introns

To calculate the sisRNA density in introns, we first concatenated all the introns for each gene. Then we divided each joined intron transcript into 100 bins. For a bin *b* with length of *L* in a certain gene *g*, we denote *b (1:L*). For each position *i* in *b* let the variable *x* at position *i* (*x*_*i*_) be 1 in case of an overlap with a sisRNA and 0 if it does not overlap. The density of sisRNAs in each bin for a certain joined intron transcript is calculated as: ${Den}_b=\sum \limits_{i=1}^L{x}_i/L$. For any given gene *j* (joined intron transcript), the set of sisRNA densities is: *G*^*j*^ = {*Den*^*j*^_*1*_, *Den*^*j*^_*2*_, …, *Den*^*j*^_*100*_}. Then the average sisRNAs density for a given bin *b* for the whole gene set (total number = *N*) is calculated as: ${Den}_b^{total}=\sum \limits_{j=1}^N{Den}_b^j/N$.

### Calculation of motif enrichment in sisRNAs and introns

To determine the enriched motifs in sisRNAs and introns, we calculated an enrichment score for each motif. To avoid the bias of sampling from the *X. tropicalis* genome, we searched the whole genome for each motif. In each scaffold, motifs were searched using MAST in MEME suite [56] with the position weight matrices (PWMs). The PWMs used in this study were collected from the TRANSFAC databases [26] in which 935 PWMs are provided and MAST was run with default parameters. For each motif *M* with the length *L* we denote *M* (*y*_*start*_*:y*_*end*_) to record the positions where the motif starts and ends*: y*_*1*_*:y*_*1*_*+L-1, y*_*2*_*: y*_*2*_*+L-1 ... y*_*N*_*:y*_*N*_*+L-1, N* being the total number of motifs in the genome. For each position *y*_*i*_*: y*_*i*_*+L-1*, if it overlapped with the examined regions, sisRNAs or introns, *x*_*i*_=1, otherwise *x*_*i*_=0. For whole sisRNAs or introns, the observed occurrences (*OCC*_*obs*_) and expected occurrences (*OCC*_*exp*_) of the motif are calculated as: ${OCC}_{obs}=\sum \limits_{i=1}^N{x}_i$ and ${OCC}_{exp}=N\times \frac{L_r}{L_g}$, where *N* is the total number of motifs in the whole genome, *L*_*r*_ is the total number of base pairs in the examined regions (sisRNAs or introns), and *L*_*g*_ is the total number of base pairs for the whole *X. tropicalis* genome. The enrichment score *E* for motif *M* was calculated as follows: $E=\frac{OCC_{obs}}{OCC_{exp}}$, where *OCC*_*obs*_ is the observed occurrences, and *OCC*_*exp*_ is the expected occurrence of motif *M* in the examined regions (sisRNAs or introns).

### Gene ontology analysis

Gene Ontology (GO) analysis was performed using DAVID (The Database for Annotation, Visualization and Integrated Discovery, http://david.abcc.ncifcrf.gov/) [57, 58]. Go terms with *P*-values < 0.01 were considered as significantly enriched.

### Evolutionary conservation analysis

Base by base PhastCons conservation scores based on an alignment and a model of neutral evolution among the seven vertebrate genomes [35] were downloaded from UCSC database (http://genome.ucsc.edu/). The seven genomes and assemblies are: zebrafish (danRer4), *X. tropicalis* (xenTro2), chicken (galGal2), opossum (monDom4), rat (rn4), mouse (mm8) and human (hg18). PhastCons scores in each sisRNA or intron were extracted for each nucleotide for ±150 bps relative to the 5′ end, midpoint, and 3′ end respectively. Values were averaged for all sisRNAs or introns.

## Supplementary information

**Additional file 1: Supplementary Figure 1.** Longer introns have more sisRNAs. (**A**) Boxplot of lengths of introns grouped by the number of bearing sisRNAs: 0 (green), 1 (orange), 2 (purple), 3 (red) and > 3 (blue). (**B**) Scatter plot of sisRNA numbers versus length of introns. (**C**) Scatter plot of sisRNA numbers versus total length of introns per gene. (**D**) Scatter plot of sisRNA numbers versus normalized intron number per Mbps per gene. **Supplementary Figure 2.** sisRNAs are specific regions of the introns. Boxplots of the comparisons of GC% (**A**), CpG density (**B**), CA|TG density (**C**), and length (**D**) of the 1st introns (green), middle introns (orange), and last introns (purple) of genes with more than 3 introns, to sisRNAs identified by refSeq (red) and Ensembl (blue) genes. **Supplementary Figure 3.** The gene ontology (GO) analysis for the genes hosting cytoplasmic sisRNAs across species. Significant enriched GO terms for genes hosting cytoplasmic sisRNAs in human red blood cells (**A**), human Hela cells (**B**), mouse red blood cells (**C**), mouse 3 T3 cells (**D**), chicken DF1 cells (**E**), and *Xenopus laevis* XTC cells (**F**).

## Data Availability

The GV and cytoplasmic RNA-seq data were published by the Gall lab, which can be found at their lab website (https://emb.carnegiescience.edu/grace/datasets). The sisRNAs and RNA-seq data in human red blood cells, Hela cells, mouse red blood cells and 3 T3 cells, chicken DF1 cells, and *X. laevis* XTC cells were obtained from [21], which have been deposited in the NCBI Sequence Read Archive with BioProject ID: PRJNA479418 (https://www.ncbi.nlm.nih.gov/bioproject/PRJNA479418/). All of the other datasets were download from UCSC web site (https://genome.ucsc.edu/). The datasets supporting the conclusions of this article are included within the article and its additional files.

## Abbreviations

5mC: 5-methylcytosine
BP: Biological process
CGI: CpG islands
DAVID: Database for annotation, visualization and integrated discovery
DC: Dendritic cells
FDR: False discovery rate
GO: Gene ontology
GV: Germinal vesicle
MACS: Model based analysis for ChIP-seq
MAST: Motif alignment and search tool in MEME suite
MBT: Midblastula transition
PhastCons: Phylogenetic analysis with space/time models for identifying evolutionarily conserved elements
PWM: Position weight matrix
RBC: Red blood cells
lncRNAs: long non-coding RNAs
miRNA: micro RNAs
sisRNAs: stable intronic sequence RNAs
mRNA: messenger RNA
tRNA: transfer RNA
rRNA: ribosomal RNA
siRNA: small interfering RNAs
piRNA: Piwi-interacting RNAs
RNA-seq: High-throughput RNA sequencing
STAT3: Signal transducer and activator of transcription 3
TFBS: transcription factor binding site

## References

[1] Jacob F, Monod J (1961). Genetic regulatory mechanisms in the synthesis of proteins. J Mol Biol.

[2] Sharp SJ, Schaack J, Cooley L, Burke DJ, Soll D (1985). Structure and transcription of eukaryotic tRNA genes. CRC Crit Rev Biochem.

[3] van Nues RW, Venema J, Rientjes JM, Dirks-Mulder A, Raue HA (1995). Processing of eukaryotic pre-rRNA: the role of the transcribed spacers. Biochem Cell Biol.

[4] Huttenhofer A, Schattner P, Polacek N (2005). Non-coding RNAs: hope or hype?. Trends Genet.

[5] Ambros V (2004). The functions of animal microRNAs. Nature.

[6] Hamilton AJ, Baulcombe DC (1999). A species of small antisense RNA in posttranscriptional gene silencing in plants. Science.

[7] Girard A, Sachidanandam R, Hannon GJ, Carmell MA (2006). A germline-specific class of small RNAs binds mammalian Piwi proteins. Nature.

[8] Perkel JM (2013). Visiting “noncodarnia”. Biotechniques.

[9] Gardner EJ, Nizami ZF, Talbot CC, Gall JG (2012). Stable intronic sequence RNA (sisRNA), a new class of noncoding RNA from the oocyte nucleus of Xenopus tropicalis. Genes Dev.

[10] Talhouarne GJ, Gall JG (2014). Lariat intronic RNAs in the cytoplasm of Xenopus tropicalis oocytes. RNA.

[11] Wahl MC, Will CL, Luhrmann R (2009). The spliceosome: design principles of a dynamic RNP machine. Cell.

[12] Domdey H, Apostol B, Lin RJ, Newman A, Brody E, Abelson J (1984). Lariat structures are in vivo intermediates in yeast pre-mRNA splicing. Cell.

[13] Chapman KB, Boeke JD (1991). Isolation and characterization of the gene encoding yeast debranching enzyme. Cell.

[14] Michaeli T, Pan ZQ, Prives C (1988). An excised SV40 intron accumulates and is stable in Xenopus laevis oocytes. Genes Dev.

[15] Kopczynski CC, Muskavitch MA (1992). Introns excised from the Delta primary transcript are localized near sites of Delta transcription. J Cell Biol.

[16] Qian L, Vu MN, Carter M, Wilkinson MF (1992). A spliced intron accumulates as a lariat in the nucleus of T cells. Nucleic Acids Res.

[17] Yang L, Duff MO, Graveley BR, Carmichael GG, Chen LL (2011). Genomewide characterization of non-polyadenylated RNAs. Genome Biol.

[18] Moss WN, Steitz JA (2013). Genome-wide analyses of Epstein-Barr virus reveal conserved RNA structures and a novel stable intronic sequence RNA. BMC Genomics.

[19] Zhang Y, Zhang XO, Chen T, Xiang JF, Yin QF, Xing YH, Zhu S, Yang L, Chen LL (2013). Circular intronic long noncoding RNAs. Mol Cell.

[20] Pek JW, Osman I, Tay ML, Zheng RT (2015). Stable intronic sequence RNAs have possible regulatory roles in Drosophila melanogaster. J Cell Biol.

[21] Talhouarne GJS, Gall JG (2018). Lariat intronic RNAs in the cytoplasm of vertebrate cells. Proc Natl Acad Sci U S A.

[22] Chan SN, Pek JW (2019). Stable Intronic sequence RNAs (sisRNAs): an expanding universe. Trends Biochem Sci.

[23] Tay ML, Pek JW (2017). Maternally inherited stable Intronic sequence RNA triggers a self-reinforcing feedback loop during development. Curr Biol.

[24] Wong JT, Akhbar F, Ng AYE, Tay ML, Loi GJE, Pek JW (2017). DIP1 modulates stem cell homeostasis in Drosophila through regulation of sisR-1. Nat Commun.

[25] Zhang Y, Liu T, Meyer CA, Eeckhoute J, Johnson DS, Bernstein BE, Nusbaum C, Myers RM, Brown M, Li W (2008). Model-based analysis of ChIP-Seq (MACS). Genome Biol.

[26] Matys V, Kel-Margoulis OV, Fricke E, Liebich I, Land S, Barre-Dirrie A, Reuter I, Chekmenev D, Krull M, Hornischer K (2006). TRANSFAC and its module TRANSCompel: transcriptional gene regulation in eukaryotes. Nucleic Acids Res.

[27] Akira S, Nishio Y, Inoue M, Wang XJ, Wei S, Matsusaka T, Yoshida K, Sudo T, Naruto M, Kishimoto T (1994). Molecular cloning of APRF, a novel IFN-stimulated gene factor 3 p91-related transcription factor involved in the gp130-mediated signaling pathway. Cell.

[28] Sen R, Baltimore D (1986). Multiple nuclear factors interact with the immunoglobulin enhancer sequences. Cell.

[29] Plaksin D, Baeuerle PA, Eisenbach L (1993). KBF1 (p50 NF-kappa B homodimer) acts as a repressor of H-2Kb gene expression in metastatic tumor cells. J Exp Med.

[30] Funk WD, Wright WE (1992). Cyclic amplification and selection of targets for multicomponent complexes: myogenin interacts with factors recognizing binding sites for basic helix-loop-helix, nuclear factor 1, myocyte-specific enhancer-binding factor 2, and COMP1 factor. Proc Natl Acad Sci U S A.

[31] Trovo-Marqui AB, Tajara EH (2006). Neurofibromin: a general outlook. Clin Genet.

[32] Kossler N, Stricker S, Rodelsperger C, Robinson PN, Kim J, Dietrich C, Osswald M, Kuhnisch J, Stevenson DA, Braun T (2011). Neurofibromin (Nf1) is required for skeletal muscle development. Hum Mol Genet.

[33] Adkins NL, Hagerman TA, Georgel P (2006). GAGA protein: a multi-faceted transcription factor. Biochem Cell Biol.

[34] Ashburner M, Ball CA, Blake JA, Botstein D, Butler H, Cherry JM, Davis AP, Dolinski K, Dwight SS, Eppig JT (2000). Gene ontology: tool for the unification of biology. The Gene Ontology Consortium. Nat Genet.

[35] Siepel A, Bejerano G, Pedersen JS, Hinrichs AS, Hou M, Rosenbloom K, Clawson H, Spieth J, Hillier LW, Richards S (2005). Evolutionarily conserved elements in vertebrate, insect, worm, and yeast genomes. Genome Res.

[36] Davidson EH (1986). Gene activity in early development.

[37] Callan HG (1986). Lampbrush chromosomes. Mol Biol Biochem Biophys.

[38] Yanai I, Peshkin L, Jorgensen P, Kirschner MW (2011). Mapping gene expression in two Xenopus species: evolutionary constraints and developmental flexibility. Dev Cell.

[39] Brocke-Heidrich K, Ge B, Cvijic H, Pfeifer G, Loffler D, Henze C, McKeithan TW, Horn F (2006). BCL3 is induced by IL-6 via Stat3 binding to intronic enhancer HS4 and represses its own transcription. Oncogene.

[40] Katoh M, Katoh M (2007). STAT3-induced WNT5A signaling loop in embryonic stem cells, adult normal tissues, chronic persistent inflammation, rheumatoid arthritis and cancer (review). Int J Mol Med.

[41] Zorn E, Nelson EA, Mohseni M, Porcheray F, Kim H, Litsa D, Bellucci R, Raderschall E, Canning C, Soiffer RJ (2006). IL-2 regulates FOXP3 expression in human CD4+CD25+ regulatory T cells through a STAT-dependent mechanism and induces the expansion of these cells in vivo. Blood.

[42] Durant L, Watford WT, Ramos HL, Laurence A, Vahedi G, Wei L, Takahashi H, Sun HW, Kanno Y, Powrie F (2010). Diverse targets of the transcription factor STAT3 contribute to T cell pathogenicity and homeostasis. Immunity.

[43] Carpenter RL, Lo HW (2014). STAT3 target genes relevant to human cancers. Cancers (Basel).

[44] Fleming J, Giresi P, Lindahl-Allen M, Krall E, Lieb J, Struhl K (2015). STAT3 acts through pre-existing nucleosome-depleted regions bound by FOS during an epigenetic switch linking inflammation to cancer. Epigenetics Chromatin.

[45] Sigova AA, Abraham BJ, Ji X, Molinie B, Hannett NM, Guo YE, Jangi M, Giallourakis CC, Sharp PA, Young RA (2015). Transcription factor trapping by RNA in gene regulatory elements. Science.

[46] Wang P, Xue Y, Han Y, Lin L, Wu C, Xu S, Jiang Z, Xu J, Liu Q, Cao X (2014). The STAT3-binding long noncoding RNA lnc-DC controls human dendritic cell differentiation. Science.

[47] Yang ZG, Awan FM, Du WW, Zeng Y, Lyu J, Wu D, Gupta S, Yang W, Yang BB (2017). The circular RNA interacts with STAT3, increasing its nuclear translocation and wound repair by modulating Dnmt3a and miR-17 function. Mol Ther.

[48] Bogdanovic O, Long SW, van Heeringen SJ, Brinkman AB, Gomez-Skarmeta JL, Stunnenberg HG, Jones PL, Veenstra GJ (2011). Temporal uncoupling of the DNA methylome and transcriptional repression during embryogenesis. Genome Res.

[49] Coulondre C, Miller JH, Farabaugh PJ, Gilbert W (1978). Molecular basis of base substitution hotspots in Escherichia coli. Nature.

[50] Bird A, Tate P, Nan X, Campoy J, Meehan R, Cross S, Tweedie S, Charlton J, Macleod D (1995). Studies of DNA methylation in animals. J Cell Sci Suppl.

[51] Burge C, Campbell AM, Karlin S (1992). Over- and under-representation of short oligonucleotides in DNA sequences. Proc Natl Acad Sci U S A.

[52] Lister R, Pelizzola M, Dowen RH, Hawkins RD, Hon G, Tonti-Filippini J, Nery JR, Lee L, Ye Z, Ngo QM (2009). Human DNA methylomes at base resolution show widespread epigenomic differences. Nature.

[53] Gardiner-Garden M, Frommer M (1987). CpG islands in vertebrate genomes. J Mol Biol.

[54] Haerty W, Ponting CP (2015). Unexpected selection to retain high GC content and splicing enhancers within exons of multiexonic lncRNA loci. RNA.

[55] Rosenbloom KR, Armstrong J, Barber GP, Casper J, Clawson H, Diekhans M, Dreszer TR, Fujita PA, Guruvadoo L, Haeussler M (2015). The UCSC genome browser database: 2015 update. Nucleic Acids Res.

[56] Bailey TL, Boden M, Buske FA, Frith M, Grant CE, Clementi L, Ren J, Li WW, Noble WS (2009). MEME SUITE: tools for motif discovery and searching. Nucleic Acids Res.

[57] Huang DW, Sherman BT, Lempicki RA (2009). Systematic and integrative analysis of large gene lists using DAVID bioinformatics resources. Nat Protoc.

[58] Huang DW, Sherman BT, Lempicki RA (2009). Bioinformatics enrichment tools: paths toward the comprehensive functional analysis of large gene lists. Nucleic Acids Res.

