# Supplementary material for: Stable intronic sequence RNAs (sisRNAs) are selected regions in introns with distinct properties
Source: BMC Genomics. 2020 Apr 7;21:287. doi: 10.1186/s12864-020-6687-9 (PMC7137253; doi:10.1186/s12864-020-6687-9)
Supplement: Supplementary file 1 — Additional file 1: Supplementary Figure 1. Longer introns have more sisRNAs. (A) Boxplot of lengths of introns grouped by the number of bearing sisRNAs: 0 (green), 1 (orange), 2 (purple), 3 (red) and > 3 (blue). (B) Scatter plot of sisRNA numbers versus length of introns. (C) Scatter plot of sisRNA numbers versus total length of introns per gene. (D) Scatter plot of sisRNA numbers versus normalized intron number per Mbps per gene. Supplementary Figure 2. sisRNAs are specific regions of the introns. Boxplots of the comparisons of GC% (A), CpG density (B), CA|TG density (C), and length (D) of the 1st introns (green), middle introns (orange), and last introns (purple) of genes with more than 3 introns, to sisRNAs identified by refSeq (red) and Ensembl (blue) genes. Supplementary Figure 3. The gene ontology (GO) analysis for the genes hosting cytoplasmic sisRNAs across species. Significant enriched GO terms for genes hosting cytoplasmic sisRNAs in human red blood cells (A), human Hela cells (B), mouse red blood cells (C), mouse 3 T3 cells (D), chicken DF1 cells (E), and Xenopus laevis XTC cells (F). [file 12864_2020_6687_MOESM1_ESM.docx]

**Supplementary Figure 1. Longer introns have more sisRNAs. (A)** Boxplot of lengths of introns grouped by the number of bearing sisRNAs: 0 (green), 1 (orange), 2 (purple), 3 (red) and >3 (blue). **(B)** Scatter plot of sisRNA numbers versus length of introns. (C) Scatter plot of sisRNA numbers versus total length of introns per gene. (D) Scatter plot of sisRNA numbers versus normalized intron number per Mbps per gene.

**Supplementary Figure 2. sisRNAs are specific regions of the introns.**

Boxplots of the comparisons of GC% **(A)**, CpG density **(B)**, CA|TG density **(C)**, and length **(D)** of the 1^st^ introns (green), middle introns (orange), and last introns (purple) of genes with more than 3 introns, to sisRNAs identified by refSeq (red) and Ensembl (blue) genes.

**Supplementary Figure 3. The gene ontology (GO) analysis for the genes hosting cytoplasmic sisRNAs across species.**

Significant enriched GO terms for genes hosting cytoplasmic sisRNAs in human red blood cells (**A**), human Hela cells (**B**), mouse red blood cells (**C**), mouse 3T3 cells (**D**), chicken DF1 cells (**E**), and *Xenopus laevis* XTC cells (**F**).
